# Interrelationships between education, occupational class and income as determinants of sickness absence among young employees in 2002–2007 and 2008–2013

**DOI:** 10.1186/s12889-015-1718-1

**Published:** 2015-04-08

**Authors:** Hilla Sumanen, Olli Pietiläinen, Jouni Lahti, Eero Lahelma, Ossi Rahkonen

**Affiliations:** Department of Public Health, University of Helsinki, Helsinki, Finland

**Keywords:** Young employees, Socioeconomic position, Education, Occupational class, Income, Sickness absence, Work disability, Gender

## Abstract

**Background:**

A low socioeconomic position (SEP) is consistently associated with ill health, sickness absence (SA) and permanent disability, but studies among young employees are lacking. We examined the interrelationships between education, occupational class and income as determinants of SA among 25-34-year-old employees. We also examined, whether the association between SEP and SA varied over time in 2002–2007 and 2008–2013.

**Methods:**

The analyses covered young, 25-34-year-old women and men employed by the City of Helsinki over the time periods 2002–2007 and 2008–2013. Four-level education and occupational class classifications were used, as well as income quartiles. The outcome measure was the number of annual SA days.

**Results:**

Education had the strongest and most consistent independent association with SA among women and men in both periods under study. Occupational class had weaker independent and less consistent association with SA. Income had an independent association with SA, which strengthened over time among the men. The interrelationships between the SEP indicators and SA were partly explained by prior or mediated through subsequent SEP indicators. Socioeconomic differences followed only partially a gradient for occupational class and also for income among men.

**Conclusions:**

Preventive measures to reduce the risk of SA should be considered, especially among young employees with a basic or lower-secondary education.

**Electronic supplementary material:**

The online version of this article (doi:10.1186/s12889-015-1718-1) contains supplementary material, which is available to authorized users.

## Background

Socioeconomic position (SEP) is consistently related to health [1]. Ill health, sickness absence (SA) and permanent disability are generally more common among those with a lower SEP [2-4]. SA reflects work ability [5], and the rates have increased in the Nordic countries especially among younger employees [6,7]. However, most research on SA concerns middle-aged or adult employees in general, and a limited number of studies focus on young employees.

SEP is a general concept that cannot be measured directly. The key indicators are education, occupational class and income, which are interrelated although each has its own effects on health as well [8,9]. Educational level relates closely to non-material resources such as knowledge and skills, and is also related to health-related behaviours [4,8,10]. It is a strong determinant of future employment and occupation as well as income [11]. We reported in our previous study that low education was strongly and independently of other two SEP indicators associated with higher SA among 25-59-year-old women and men [4].

Occupational class reflects physical and psychosocial working conditions, and these affect health and SA in many ways [12,13]. Lower occupational classes have more SA than those in higher classes [14-18]. There is some evidence that this association exists among younger employees as well [19], but further analysis is needed. It has been found that occupational class is an independent determinant of medically confirmed SA among Finnish employees [4].

Income reflects access to material resources [8] such as those required to buy healthier food, and allows access to services and health-related leisure activities [11]. A higher income is strongly associated with a reduced risk of SA in the Nordic countries [1,17,20]. However, income is the SEP indicator that may change most rapidly over time [11]. We found in our previous study with broader age range that education and occupational class largely explained the association between income and SA [4,9].

**Figure 1:**
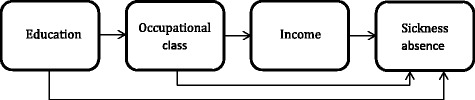
**Assumed interrelationships between the socioeconomic determinants of sickness absence (adapted from Lahelma et al.** [9]**).**

Examining the interrelationships between the key SEP indicators and SA gives a comprehensive picture of the association between SEP and SA and of the temporal pathways between the indicators (Figure 1). The effect of education on SA is assumed to be partly mediated through the succeeding occupational class and further through income. The effect of occupational class on SA is assumed to be partly explained by preceding education or to be mediated through income. The effect of income on SA is assumed to be partly explained by education and occupational class [9,21,22]. The interrelationships between education, occupational class and income with regard to SA have previously been studied among adult Finnish employees [4], but not among young employees. In order to fill this gap, we examined the interrelationships between education, occupational class and income as determinants of sickness absence in 25–34 year old female and male employees. Given the changes in SA among young employees during the last decade [6,7] and the economic downturn that started in Finland in 2008, we also examined, whether the associations between the key SEP indicators and SA varied over time in 2002–2007 and 2008–2013.

## Methods

### Data

This study is part of the Helsinki Health Study on health and wellbeing among employees of the City of Helsinki, Finland. Helsinki is the capital of Finland, and the municipality is the largest employer in the country. Its main areas of operation include healthcare, education, social welfare services, public transport, culture, construction and technical services. All employees are covered by the same personnel administration and registration systems, policies and occupational health care [23].

We used the City of Helsinki’s personnel and SA registers to obtain individual-level information on its employees’ SA, occupational class, income and socio-demographic factors. The registers cover all employees, their work contracts and SA spells to an accuracy of one day [17]. Information on education was obtained from Statistics Finland’s register of completed education and degrees [24] and was linked to the City of Helsinki register data.

### Methods

All permanently and temporarily employed staff from the periods 2002–2007 and 2008–2013 and aged 25–34 were initially included in the analyses. Employees working less than 28 hours a week and those with no registered income (being on leave or for other reasons) were excluded. After these exclusions 22,046 women and 7,155 men were included in the analyses (Table 1). The ethics committees of the Department of Public Health, the University of Helsinki and the health authorities of the City of Helsinki approved the study.

**Table 1:** **Distribution of socioeconomic indicators and sickness absence days/100 person years, by gender in 2002–2007 and 2008–2013**

		**Women (N = 22046)**		**Men (N = 7155)**	
		**%**	**Sickness absence days/100 person years**	**%**	**Sickness absence days/100 person years**
**2002-2007**					
**Education**	Higher	10	723	9	524
	Upper secondary	39	1329	23	817
	Lower secondary	44	1849	54	1281
	Basic	7	2602	14	1953
**Occupational class**	Managers and professionals	13	811	14	555
	Semi-professionals	29	1406	18	885
	Routine non-manuals	48	1819	30	1367
	Manual workers	10	1898	38	1460
**Individual income**	Highest	25	1023	25	708
	Second quartile	25	1485	25	1222
	Third quartile	25	1918	25	1376
	Lowest	25	1998	25	1582
**2008-2013**					
**Education**	Higher	14	829	12	546
	Upper secondary	40	1304	24	832
	Lower secondary	41	2036	54	1222
	Basic	5	2576	11	1789
**Occupational class**	Managers and professionals	14	825	17	496
	Semi-professionals	33	1363	19	898
	Routine non-manuals	44	1988	27	1371
	Manual workers	8	1834	38	1312
**Individual income**	Highest	25	1017	25	551
	Second quartile	25	1397	25	1118
	Third quartile	25	1970	25	1391
	Lowest	25	2138	25	1472

#### Sickness absence (SA)

We examined the total annual numbers of SA days in this study. All interruptions in working for reasons other than the employee’s own illnesses, such as child’s illness or work injury absences were excluded from the analyses. Overlapping SA spells were combined, so that all SA days were counted only once.

#### Education

Education was classified on four levels annually according to the highest qualification [24]: basic education, (comprehensive school), lower secondary (upper-secondary school, vocational school), upper secondary (a bachelor’s degree from a university or institution of applied sciences) and higher education (a master’s or doctoral degree).

#### Occupational class

Occupational class was assigned to one of four categories based on the job title: managers and professionals such as teachers and physicians; semi-professionals such as nurses and foremen; routine non-manual workers, such as clerical employees and child minders; and manual workers such as technical and cleaning staff [8].

#### Income

The employees were placed annually in four groups based on income quartiles covering their own monthly salary related to their employment with the City of Helsinki, women and men separately. The measure covers only individual wages or salaries and does not include income from other sources such as secondary employment, investments or income transfers.

### Statistical methods

First we calculated the numbers of SA days per 100-person-years for each level of education, occupational class and income quartile.

We then analysed the associations of education, occupational class and income with SA days using generalised linear mixed models via penalised quasi-likelihood Poisson regression [25]. We preferred quasi-likelihood Poisson regression to ordinary Poisson regression due to over-dispersion in the data. Individual-specific random intercept was used to take yearly repeated measurements into account. Different lengths of employment contracts were taken into account by using logarithm of days employed as the offset.

First, models with each socioeconomic indicator adjusted for age and measurement year were fitted to obtain the age- and period-adjusted effect of each socioeconomic indicator, hereafter referred to as the age-adjusted associations. Then different models with all combinations of the socioeconomic indicators adjusted for age and measurement year were fitted to analyse the relations of mediation or explanation between the socioeconomic indicators as determinants of SA. For example, if the association of education with SA is attenuated when occupational class or income is added to the model, they are considered to mediate the original association. Furthermore, if the association of income with SA is attenuated when education or occupational class is added to the model, they are considered to explain the original association. The effect of each socioeconomic indicator independent from the other two socioeconomic indicators was assessed from the full model where all socioeconomic indicators were adjusted for simultaneously.

Separate analyses were conducted among women and men, and in 2002–2007 and 2008–2013. We used the glmmPQL function in the MASS package [26], R statistical software version 2.13.0, for the analyses.

## Results

There were more women than men in the data (Table 1). Lower secondary was the most common educational level among both women (44% in 2002–2007, 41% in 2008–2013) and men (54% and 54%, respectively). Routine non-manual workers (48% and 44%, respectively) comprised the largest occupational class among the women, whereas manual workers (38%, 38%) were the largest class among the men.

SA was higher in the lower socioeconomic positions among both women and men: those with a basic education, for example, had approximately 3.5 times more SA than their more highly educated counterparts (Table 1).

### Women

There was an educational gradient in SA among the women in 2002–2007: those with a basic (age-adjusted RR 3.46, CI 3.12, 3.84), a lower-secondary (age-adjusted RR 2.36, CI 2.17, 2.56) and an upper-secondary (age-adjusted RR 1.80 CI 1.66, 1.96) education had a higher risk of SA than those with a higher education (Table 2). The age-adjusted association attenuated following adjustment for occupational class and, to a lesser extent, for income. The gradient and significant differences remained after mutual adjustment.

**Table 2:** Rate ratio of sickness absence days per 100 person years by socioeconomic indicators from different regression models, women*

		**Gross effect**	**Occupation + education**	**Occupation + income**	**Education + income**	**Occupation + education + income**
**2002–2007**						
**Education**	Higher	1	1		1	1
	Upper secondary	1.80 (1.66, 1.96)	1.35 (1.22, 1.50)		1.66 (1.52, 1.81)	1.33 (1.20, 1.48)
	Lower secondary	2.36 (2.17, 2.56)	1.82 (1.64, 2.03)		1.96 (1.79, 2.16)	1.70 (1.53, 1.90)
	Basic	3.46 (3.12, 3.84)	2.71 (2.39, 3.08)		2.81 (2.50, 3.15)	2.48 (2.18, 2.82)
**Occupational class**	Managers and professionals	1	1	1		1
	Semi-professionals	1.82 (1.68, 1.96)	1.54 (1.40, 1.68)	1.66 (1.53, 1.79)		1.45 (1.32, 1.59)
	Routine non-manuals	2.13 (1.98, 2.29)	1.45 (1.32, 1.59)	1.63 (1.49, 1.78)		1.25 (1.13, 1.38)
	Manual workers	2.11 (1.93, 2.31)	1.30 (1.17, 1.46)	1.51 (1.36, 1.68)		1.08 (0.96, 1.22)
**Individual income**	Highest	1		1	1	1
	Second quartile	1.35 (1.28, 1.42)		1.19 (1.12, 1.25)	1.16 (1.10, 1.22)	1.14 (1.08, 1.20)
	Third quartile	1.59 (1.51, 1.67)		1.38 (1.29, 1.47)	1.26 (1.18, 1.34)	1.26 (1.18, 1.35)
	Lowest	1.74 (1.65, 1.84)		1.52 (1.42, 1.63)	1.31 (1.23, 1.40)	1.35 (1.25, 1.45)
**2008–2013**						
**Education**	Higher	1	1		1	1
	Upper secondary	1.58 (1.47, 1.69)	1.27 (1.17, 1.38)		1.44 (1.34, 1.55)	1.25 (1.15, 1.36)
	Lower secondary	2.27 (2.13, 2.43)	1.76 (1.61, 1.93)		1.86 (1.72, 2.01)	1.66 (1.52, 1.82)
	Basic	2.82 (2.56, 3.10)	2.24 (2.00, 2.51)		2.25 (2.03, 2.50)	2.07 (1.85, 2.32)
**Occupational class**	Managers and professionals	1	1	1		1
	Semi-professionals	1.66 (1.55, 1.77)	1.41 (1.30, 1.53)	1.50 (1.39, 1.61)		1.32 (1.21, 1.44)
	Routine non-manuals	2.21 (2.07, 2.36)	1.49 (1.37, 1.63)	1.67 (1.53, 1.81)		1.28 (1.16, 1.41)
	Manual workers	1.98 (1.81, 2.16)	1.26 (1.13, 1.40)	1.40 (1.26, 1.56)		1.04 (0.92, 1.17)
**Individual income**	Highest	1		1	1	1
	Second quartile	1.37 (1.30, 1.44)		1.16 (1.10, 1.23)	1.18 (1.12, 1.25)	1.12 (1.06, 1.19)
	Third quartile	1.70 (1.62, 1.79)		1.36 (1.27, 1.45)	1.29 (1.22, 1.37)	1.23 (1.15, 1.32)
	Lowest	1.85 (1.76, 1.95)		1.50 (1.40, 1.61)	1.36 (1.28, 1.45)	1.33 (1.24, 1.43)

*Adjusted for age and measurement year.

The age-adjusted association between occupational class and SA followed a partial gradient as routine non-manual workers (RR 2.13, CI 1.98, 2.29) had almost the same risk of SA than manual workers (RR 2.11, CI 1.93, 2.31). Adjusting for education attenuated the association, especially among the lower occupational classes. Adjusting for income attenuated the association less, but only among the lower occupational classes, bringing them close to the semi-professionals (RR 1.66, CI 1.53, 1.79). Occupational class had an independent association with SA in the full model. Especially semi-professionals had an elevated risk (RR 1.45, CI 1.32, 1.59).

The age-adjusted association with income and SA followed a gradient, as the fourth (RR 1.74, CI 1.65, 1.84) income quartile had the highest risks of SA. Adjusting for occupational class attenuated the association slightly less than adjusting for education, but the differences were small. Income had an independent association with SA in the full model (RR 1.14, CI 1.08, 1.20 for the second, RR 1.26, CI 1.18, 1.35 for the third and RR 1.35, CI 1.25, 1.45 for the fourth quartile).

The age-adjusted association between education and SA in 2008–2013 was broadly similar to that in the first period, only those with a basic education showing a weaker association. In addition, adjusting for occupational class or income attenuated the association similarly as in the first period. In the full model the association was broadly similar as in the first period among those with a lower-secondary (RR 1.66, CI 1.52, 1.82) or upper-secondary (RR 1.25, CI 1.15, 1.36) education, but was weaker for those with a basic education (RR 2.07, CI 1.85, 2.32).

The age-adjusted association between occupational class and SA was also broadly similar in 2008–2013 compared to the first period. The attenuation following adjustment for education or income remained similar as in the first period. Occupational class in 2008–2013 also had a weak independent association with SA in the full model and followed a partial gradient (RR 1.32, CI 1.21, 1.44 for semi-professionals, RR 1.28, CI 1.16, 1.41 for routine non-manual workers and RR 1.04, CI 0.92, 1.17 for manual workers).

The age-adjusted association between income and SA was similar as in the first period. In 2008–2013 income had an independent association with SA in the full model similarly to the first period.

### Men

There was a clear gradient between education and SA among the men in 2002–2007 (age-adjusted RR 3.70, CI 3.05, 4.48 for basic, age-adjusted RR 2.50, CI 2.10, 2.98 for lower-secondary and age-adjusted RR 1.76, CI 1.46, 2.12 for upper-secondary education) (Table 3). Adjusting for occupational class attenuated the association, but adjusting for income attenuated it only slightly further in the full model.

**Table 3:** Rate ratio of sickness absence days per 100 person years by socioeconomic indicators from different regression models, men*

		**Gross effect**	**Occupation + education**	**Occupation + income**	**Education + income**	**Occupation + education + income**
**2002–2007**						
**Education**	Higher	1	1		1	1
	Upper secondary	1.76 (1.46, 2.12)	1.34 (1.08, 1.66)		1.46 (1.21, 1.77)	1.31 (1.06, 1.62)
	Lower secondary	2.50 (2.10, 2.98)	1.68 (1.36, 2.07)		1.81 (1.49, 2.19)	1.57 (1.27, 1.94)
	Basic	3.70 (3.05, 4.48)	2.41 (1.92, 3.03)		2.56 (2.08, 3.16)	2.23 (1.77, 2.81)
**Occupational class**	Managers and professionals	1	1	1		1
	Semi-professionals	1.54 (1.32, 1.79)	1.29 (1.09, 1.53)	1.35 (1.15, 1.58)		1.18 (0.99, 1.40)
	Routine non-manuals	2.27 (1.97, 2.61)	1.73 (1.47, 2.04)	1.70 (1.43, 2.02)		1.42 (1.18, 1.71)
	Manual workers	2.44 (2.13, 2.81)	1.72 (1.45, 2.03)	1.70 (1.43, 2.03)		1.35 (1.11, 1.63)
**Individual income**	Highest	1		1	1	1
	Second quartile	1.74 (1.57, 1.92)		1.39 (1.23, 1.57)	1.49 (1.34, 1.66)	1.33 (1.17, 1.50)
	Third quartile	1.88 (1.68, 2.09)		1.42 (1.24, 1.63)	1.49 (1.32, 1.69)	1.30 (1.13, 1.49)
	Lowest	2.25 (2.01, 2.52)		1.67 (1.43, 1.94)	1.70 (1.50, 1.94)	1.49 (1.28, 1.74)
**2008–2013**						
**Education**	Higher	1	1		1	1
	Upper secondary	1.58 (1.36, 1.84)	1.12 (0.95, 1.32)		1.25 (1.07, 1.46)	1.08 (0.91, 1.27)
	Lower secondary	2.29 (1.99, 2.64)	1.42 (1.20, 1.68)		1.45 (1.24, 1.69)	1.25 (1.06, 1.48)
	Basic	3.36 (2.84, 3.96)	2.05 (1.69, 2.48)		2.02 (1.69, 2.42)	1.76 (1.45, 2.13)
**Occupational class**	Managers and professionals	1	1	1		1
	Semi-professionals	1.88 (1.65, 2.15)	1.70 (1.47, 1.96)	1.48 (1.29, 1.70)		1.41 (1.22, 1.63)
	Routine non-manuals	2.83 (2.50, 3.21)	2.30 (1.99, 2.66)	1.75 (1.50, 2.03)		1.58 (1.34, 1.85)
	Manual workers	2.52 (2.22, 2.84)	1.93 (1.66, 2.23)	1.42 (1.22, 1.66)		1.25 (1.06, 1.48)
**Individual income**	Highest	1		1	1	1
	Second quartile	1.94 (1.76, 2.14)		1.59 (1.42, 1.78)	1.73 (1.56, 1.92)	1.54 (1.38, 1.73)
	Third quartile	2.61 (2.36, 2.89)		2.10 (1.85, 2.38)	2.21 (1.97, 2.47)	1.97 (1.73, 2.24)
	Lowest	2.43 (2.19, 2.70)		2.07 (1.81, 2.37)	1.98 (1.76, 2.23)	1.89 (1.65, 2.17)

*Adjusted for age and measurement year.

The age-adjusted association between occupational class and SA followed a gradient (RR 2.44, CI 2.13, 2.81 for manual workers, RR 2.27, CI 1.97, 2.61 for routine non-manual workers and RR 1.54, CI 1.32, 1.79 for semi-professionals). Adjusting for education attenuated the association similarly as adjusting for income. Occupational class had a weak independent association with SA in the full model and followed a partial gradient.

The age-adjusted association between income and SA followed a gradient. Adjusting for occupational class attenuated the association similarly as adjusting for education. In the full model income had an independent association with SA and followed a partial gradient (RR 1.33, CI 1.17, 1.50 for the second, RR 1.30, CI 1.13, 1.49 for the third and RR 1.49, CI 1.28, 1.74 for the fourth quartile).

The age-adjusted association between education and SA among the men was slightly weaker in 2008–2013 than in the first period. Adjusting for occupational class or income attenuated the association similarly as in the first period. In the full model, the association between education and SA was considerably weaker than in the first period (RR 1.76, CI 1.45, 2.13 for basic and RR 1.25, CI 1.06, 1.48 for lower-secondary education) and disappeared for upper-secondary education.

Unlike in the first period, the age-adjusted association between occupational class and SA followed a partial gradient in 2008–2013, and adjusting for income attenuated the association more than adjusting for education. The association between occupational class and SA in the full model was stronger than in the first period and followed a partial gradient.

In the second period, the age-adjusted association between income and SA followed a partial gradient and was strong in the third (RR 2.61, CI 2.36, 2.89) and fourth quartile (RR 2.43, CI 2.19, 2.70) in particular. Adjusting for education or occupational class somewhat attenuated the association. In 2008–2013 income had stronger independent association with SA in the full model than in the first period.

## Discussion

Our aim was to examine the interrelationships between three key socioeconomic indicators, education, occupational class and income, as determinants of sickness absence among young women and men. The investigation covered two time periods, 2002–2007 and 2008–2013. The main findings were as follows. 1) Education had the strongest and most consistent independent association with SA among women and men in both periods under study. 2) Occupational class had weaker independent and less consistent association with SA. 3) Income had an independent association with SA, which strengthened over time among the men. 4) Socioeconomic differences followed only partially a gradient for occupational class and also for income among men.

The association between education and SA was partly mediated through occupational class, and that between occupational class and SA was partly explained by education in both genders. The role of income mediating these associations was smaller, among women in particular. We identified interrelationships between the studied socioeconomic indicators and SA in our previous study among 25-59-year-old employees [4], and expected to do so among the young employees in this study: education and occupational class were both strong independent determinants of SA in our previous study, in our present study, also income had independent association with SA.

The interrelationship between education and occupational class may be partly attributable to the fact that educational qualifications are needed for many occupations in the public sector. The more highly educated are also more knowledgeable about health-related issues and are better equipped to make healthier lifestyle choices [11], hence a higher SA among those with a lower education was to be expected.

The association between a low occupational class and SA [4,15,18,19] might reflect the fact that employees in manual occupations have more physically demanding jobs, which may affect their work ability and contribute to disability. On the one hand, managers and professionals may have more flexibility in their work-related tasks, and may be able to work inside or outside their place of employment when they are ill, for example [19]. On the other hand, employees in the higher occupational classes may have more complex and mentally demanding jobs [27]. However, physical and mental working conditions might not yet affect the health of young employees, who have been exposed to them for a relatively short time, and the health-related effects may take longer to appear. This could explain the weaker association between occupational class and SA, compared to education.

The gradient in the age-adjusted association between occupational class and SA was more visible in the present study, and changed in the full models. Education partly explained the association, especially with regard to routine non-manual and manual workers. This is understandable given that employees in the municipal sector are unlikely to reach the upper occupational classes without a higher-level degree.

However, those with a basic education had a consistently increased risk of SA, which is in line with the results of previous studies on the association between education and SA [4,10,17]. Employees without qualifications may have minor jobs and poor working conditions, and consequent ill health. It is also possible that some of the young employees with a basic education in our study were still in the process of studying secondary education, as many occupations require qualifications. If so, one could speculate that the risk of SA is higher, as the burden of studying and working might affect health through reduced time for sleep, exercise and recovery from work [28-32].

There was a strong independent association between income and SA among the men particularly during the second period, and also independent association among the women. However, given that income typically follows a curvilinear trajectory with age, it may not be the most reliable indicator of SEP among young adults [11]. We used individual rather than household income. When analysing only employees with full weekly working hours, the association between income and SA was very similar (see Additional file 1: Table S4 & Additional file 2: Table S5). In our previous study with broader age range, education and occupational class explained the effect of income among both women and men [4]. Given that education largely determines the level of income in female-dominated occupations (i.e. nurses and teachers) in the municipal sector, it is understandable that other two socioeconomic indicators explain some of the association between income and SA. However, our results differ and this phenomenon requires further study.

### Methodological considerations

This study was based on large number of young employees of the City of Helsinki and extensive SA registers held by the employer, providing a reliable and comprehensive data source. However, the employer’s registers lack further information on the participants.

We linked the employer’s register to Statistics Finland’s register of completed education and degrees, which is an accurate national register that includes the highest degree or the most recent qualification and is updated annually. Information on degrees and qualifications comes straight from educational institutions and government agencies.

We were able to study the interrelationships between education, occupational class and income as determinants of SA in two different time periods. Recurrence of the associations found in the both studied periods strengthens our results. However, there are also other factors potentially affecting SA, such as individual characteristics, personal lifestyles and health and also numerous work characteristics, for example psychosocial, work content and physical working exposures [30]. Due to the limited nature of the register data we were unable to assess the contribution of these different factors to the socioeconomic differences in SA. The participants in our study were young municipal employees of the City of Helsinki, the largest employer in Finland. The results could be generalised with caution to the Finnish municipal sector, but not to the labour force in general.

## Conclusions

Education, occupational class and income appear to be independent determinants of SA among young women and men. However, the associations between these SEP indicators and SA are partly explained by prior or mediated through subsequent SEP indicators. There were clear socioeconomic differences among the young employees in the study, but the socioeconomic gradient was not fully consistent throughout the analyses.

Preventive measures to reduce the risk of SA should be considered, especially among young employees with a basic or lower secondary education and those in lower occupational positions or with low income. Early intervention tools which utilize resources efficiently should be developed for maintain and preserve work ability and prevent SA among these high risk groups. Work places and job demands across different occupations should be improved to the direction where coping with work could be more easily matched with employees work ability.

## Additional files

Additional file 1: Table S4. Rate ratio of sickness absence days per 100 person years by socioeconomic indicators from different regression models, women. Additional file 2: Table S5. Rate ratio of sickness absence days per 100 person years by socioeconomic indicators from different regression models, men.

## Abbreviations

SEP: Socioeconomic position
SA: Sickness absence
