# Supplementary material for: Interrelationships between education, occupational class and income as determinants of sickness absence among young employees in 2002–2007 and 2008–2013
Source: BMC Public Health. 2015 Apr 8;15:332. doi: 10.1186/s12889-015-1718-1 (PMC4393569; doi:10.1186/s12889-015-1718-1)
Supplement: Additional file 1: Table S4. — Rate ratio of sickness absence days per 100 person years by socioeconomic indicators from different regression models, women. [file 12889_2015_1718_MOESM1_ESM.pdf]

Table 4: Rate ratio of sickness absence days per 100 person years by socioeconomic indicators from different regression models, women *						
		Gross effect	Occupation + education	Occupation + income	Education + income	Occupation + Education + Income
2002–2007						
**Education**	Higher	1	1		1	1
	Upper secondary	1.86 (1.70, 2.03)	1.40 (1.26, 1.56)		1.70 (1.55, 1.86)	1.37 (1.23, 1.53)
	Lower secondary	2.42 (2.22, 2.64)	1.90 (1.70, 2.13)		1.98 (1.80, 2.18)	1.74 (1.56, 1.95)
	Basic	3.56 (3.19, 3.96)	2.84 (2.49, 3.24)		2.81 (2.49, 3.16)	2.53 (2.22, 2.89)
**Occupational class**	Managers and professionals	1	1	1		1
	Semi-professionals	1.84 (1.70, 1.99)	1.53 (1.40, 1.69)	1.66 (1.53, 1.80)		1.44 (1.30, 1.58)
	Routine non-manuals	2.15 (1.99, 2.32)	1.43 (1.30, 1.57)	1.55 (1.42, 1.70)		1.19 (1.08, 1.32)
	Manual workers	2.10 (1.92, 2.31)	1.27 (1.13, 1.42)	1.39 (1.25, 1.55)		1.01 (0.89, 1.14)
**Individual income**	Highest	1		1	1	1
	Second quartile	1.37 (1.30, 1.44)		1.23 (1.16, 1.30)	1.18 (1.12, 1.25)	1.17 (1.11, 1.24)
	Third quartile	1.60 (1.52, 1.69)		1.45 (1.35, 1.55)	1.27 (1.19, 1.35)	1.30 (1.21, 1.40)
	Lowest	1.82 (1.72, 1.93)		1.68 (1.56, 1.81)	1.36 (1.27, 1.46)	1.45 (1.34, 1.57)
2008–2013						
**Education**	Higher	1	1		1	1
	Upper secondary	1.55 (1.44, 1.66)	1.23 (1.13, 1.34)		1.39 (1.29, 1.50)	1.21 (1.11, 1.32)
	Lower secondary	2.26 (2.10, 2.42)	1.73 (1.58, 1.89)		1.78 (1.63, 1.93)	1.60 (1.46, 1.76)
	Basic	2.74 (2.48, 3.02)	2.13 (1.90, 2.40)		2.09 (1.87, 2.33)	1.93 (1.72, 2.17)
**Occupational class**	Managers and professionals	1	1	1		1
	Semi-professionals	1.66 (1.54, 1.78)	1.44 (1.33, 1.57)	1.46 (1.35, 1.58)		1.32 (1.21, 1.45)
	Routine non-manuals	2.20 (2.05, 2.35)	1.52 (1.40, 1.66)	1.54 (1.41, 1.69)		1.24 (1.13, 1.38)
	Manual workers	1.99 (1.82, 2.18)	1.31 (1.17, 1.46)	1.29 (1.15, 1.44)		1.01 (0.90, 1.14)
**Individual income**	Highest	1		1	1	1
	Second quartile	1.40 (1.33, 1.47)		1.21 (1.14, 1.28)	1.23 (1.16, 1.29)	1.16 (1.10, 1.23)
	Third quartile	1.74 (1.66, 1.84)		1.45 (1.35, 1.56)	1.34 (1.26, 1.43)	1.30 (1.20, 1.39)
	Lowest	1.96 (1.85, 2.07)		1.66 (1.54, 1.80)	1.45 (1.35, 1.55)	1.44 (1.33, 1.57)

* Only full-time employees, adjusted for age and measurement year
